# PET imaging of ^68^Ga-NODAGA-RGD, as compared with ^18^F-fluorodeoxyglucose, in experimental rodent models of engrafted glioblastoma

**DOI:** 10.1186/s13550-018-0405-5

**Published:** 2018-06-15

**Authors:** Sibel Isal, Julien Pierson, Laetitia Imbert, Alexandra Clement, Charlotte Collet, Sophie Pinel, Nicolas Veran, Aurélie Reinhard, Sylvain Poussier, Guillaume Gauchotte, Steeven Frezier, Gilles Karcher, Pierre-Yves Marie, Fatiha Maskali

**Affiliations:** 1 Department of Nuclear Medicine, CHRU-Nancy, F-54000 Nancy, France; 2 Nancyclotep Molecular and Experimental Imaging Platform, CHRU-Nancy, Lorraine University, Nancy, F-54000 France; 3 Lorraine University, INSERM, IADI, UMR 1254, 5, rue du MORVAN, F-54000 Nancy, France; 4 Lorraine University, CNRS, CRAN, UMR 7039, 9, avenue de la forêt de Haye, 54000 Nancy, France; 5 Department of Pathology, CHRU-Nancy, Université de Lorraine, 9, avenue de la forêt de Haye, F-54000 Nancy, France; 6 Lorraine University, INSERM, NGERE, UMR 954, 9, avenue de la forêt de Haye, F-54000 Nancy, France; 7 Lorraine University, INSERM, DCAC, UMR 1116, 9, avenue de la forêt de Haye, F-54000 Nancy, France; 8 POSIFIT, Nancyclotep Molecular and Experimental Imaging Platform, 5, rue du MORVAN, Nancy, F-54000 France

**Keywords:** ^68^Ga-NODAGA-RGD, ^18^F-FDG, PET, αvβ3 integrin, Glioblastoma

## Abstract

**Background:**

Tracers triggering αvβ3 integrins, such as certain RGD-containing peptides, were found promising in previous pilot studies characterizing high-grade gliomas. However, only limited comparisons have been performed with current PET tracers. This study aimed at comparing the biodistribution of ^18^F-fluorodeoxyglucose (^18^F-FDG) with that of ^68^Ga-NODAGA-RGD, an easily synthesized monomeric RGD compound with rapid kinetics, in two different rodent models of engrafted human glioblastoma.

**Methods:**

Nude rodents bearing human U87-MG glioblastoma tumor xenografts in the flank (34 tumors in mice) or in the brain (5 tumors in rats) were analyzed. Kinetics of ^68^Ga-NODAGA-RGD and of ^18^F-FDG were compared with PET imaging in the same animals, along with additional autohistoradiographic analyses and blocking tests for ^68^Ga-NODAGA-RGD.

**Results:**

Both tracers showed a primary renal route of clearance, although with faster clearance for ^68^Ga-NODAGA-RGD resulting in higher activities in the kidneys and bladder. The tumor activity from ^68^Ga-NODAGA-RGD, likely corresponding to true integrin binding (i.e., suppressed by co-injection of a saturating excess of unlabeled RGD), was found relatively high, but only at the 2^nd^ hour following injection, corresponding on average to 53% of total tumor activity. Tumor uptake of ^68^Ga-NODAGA-RGD decreased progressively with time, contrary to that of ^18^F-FDG, although ^68^Ga-NODAGA-RGD exhibited 3.4 and 3.7-fold higher tumor-to-normal brain ratios on average compared to ^18^F-FDG in mice and rat models, respectively. Finally, ex-vivo analyses revealed that the tumor areas with high ^68^Ga-NODAGA-RGD uptake also exhibited the highest rates of cell proliferation and αv integrin expression, irrespective of cell density.

**Conclusions:**

^68^Ga-NODAGA-RGD has a high potential for PET imaging of glioblastomas, especially for areas with high integrin expression and cell proliferation, although PET recording needs to be delayed until the 2^nd^ hour following injection in order to provide sufficiently high integrin specificity.

**Electronic supplementary material:**

The online version of this article (10.1186/s13550-018-0405-5) contains supplementary material, which is available to authorized users.

## Background

High-grade gliomas constitute the most frequent cause of brain primary tumors and remain associated with a very poor prognosis under treatment [1]. Imaging plays a key role in their diagnosis and medical monitoring. Although magnetic resonance imaging is the first-line technique, positron emission tomography (PET) is increasingly used in this setting [2]. The diagnostic information obtained with ^18^F-fluorodeoxyglucose (^18^F-FDG) is somewhat variable whereas amino acid PET tracers, such as ^11^C-methionine, ^18^F-FDOPA, and ^18^F-FET, allow a more accurate, tumor characterization [3], which may also be the case for more recent radiotracers developed for PET imaging of αvβ3 integrins [4, 5].

These integrins are currently expressed in glioma cells and in neo-vessel endothelial cells, in conjunction with tumor-related angiogenic processes [6]. The expression of αvβ3 integrins increases with the malignancy grade of the glioma and appears particularly important in high-grade gliomas where αvβ3 integrins may facilitate tumor progression [7]. Most of the radiotracers that bind these integrins contain one or several tripeptide Arg-Gly-Asp (RGD) amino acid sequences owing to their high affinity for αvβ3 integrins [4, 5, 8–11]. While some of these RGD-containing peptides have been labeled with Fluor-18, their synthesis appears to be too complex for routine applications despite their high diagnostic potential [9, 12].

Other RGD-containing peptides, labeled with ^68^Ga through a metal complexation process, are much easier to synthesize [5, 10, 11]. This is notably the case of cyclic RGD peptides conjugated with a macrocyclic chelator, such as the dimeric ^68^Ga-NOTA-PRGD2 peptide, which was successfully tested in human gliomas and exhibiting a superiority to ^18^F-FDG in differentiating high- from low-grade gliomas [5]. However, such comparison with ^18^F-FDG has never been undertaken to date for ^68^Ga-NODAGA-RGD, a monomeric peptide containing only a single cyclic RGD (c(RGDyK)) and involving NODAGA as the chelating group [13]. Although the affinity for αvβ3 integrins is lower for monomeric than for multimeric RGD-containing tracers, this is seemingly counterbalanced by faster kinetics and diffusion rates, owing to their much smaller size and leading to a favorable biodistribution for tumor imaging, as shown in a previous pre-clinical study [10].

In light of the above, the present study aimed at comparing the biodistribution of ^18^F-FDG to that of ^68^Ga-NODAGA-RGD, a monomeric RGD-containing tracer with ease of synthesis and rapid kinetics, in two different rodent models of engrafted human glioblastoma.

## Methods

### Production and quality control of ^68^Ga-NODAGA-RGD

NODAGA-RGD was purchased from ABX GmbH (Hamburg, Germany). The ^68^Ga-labeling of NODAGA-RGD was performed using a mAIO synthesizer (Trasis, Ans, Belgium) with removable cassettes. The ^68^Ge/^68^Ga generator (IGG-100; Eckert & Ziegler Europe, 370 MBq) was eluted with 5 mL of a 0.1 M HCl solution. The eluate was used without pre-purification. NODAGA-RGD (10 μg, 10 nmol) was dissolved at 30 °C for 5 min in 1 mL of 0.7 M NaOAc buffer. The resulting product, ^68^Ga-NODAGA-RGD, was purified on a SepPak^®^ C18 light cartridge, eluted with 50% (0.5/0.5 *v*/*v*) ethanol and completed with a 0.9% saline solution in order to reach a total volume of 5 mL. High-performance liquid chromatography (HPLC) analyses were performed on a Waters system (Milford, Massachusetts, USA) equipped with a 2695eb pump, an auto sampler injector, a UV spectrometer (2998 PDA detector), and a Berthold LB-500 radioactivity detector, controlled by the Empower software.

HPLC conditions consisted of an ACE C18 column (AIT, 150 × 3 mm, 3 μm), flow rate 0.5 mL/min at 25 °C, acetonitrile (CH3CN)/H_2_O/0.1% trifluoroacetic acid (TFA) gradient, 0–2 min 0% CH3CN, and 2–16 min 0–40% CH3CN.

### Animal models and experimental plan

All protocols were approved by the Lorraine Ethics Committee N°68 according to Guidelines of Animal Care and Use (APAFIS no. 2456-2015102609499994).

Tumor xenografts were obtained in mice flanks as described previously [14]. Six-week-old female immunodeficient (nu/nu) NMRI mice, weighing 20–25 g, were obtained from Janvier Laboratories (Le Genest Saint Isle, France) and housed in ventilated cages including filter tops with an ad libitum access to food and water. Two xenograft tumors were induced in all mice through subcutaneous flank injections of 2.10^6^ of human U87-MG cells. Tumor growth was assessed twice weekly, according to tumor volume (in mm^3^) calculated with the formula d2 × D/2, where d and D represent the shortest and longest diameters, respectively, calculated in millimeter with a caliper (decrescent PRC, EDVC, France).

Seventeen of these mice, with tumor volumes ranging from 150 to 750 mm^3^, were referred to the experimental protocol involving two consecutive PET recording procedures at a 48-h interval, one performed after the injection of ^68^Ga-NODAGA-RGD and the other after the injection of ^18^F-FDG. In order to prevent any order effect, the ^68^Ga-NODAGA-RGD PET was performed first in half of the animals and second (after ^18^F-FDG injection) in the other half. At the end of the 2nd PET recording, the animals were sacrificed by inhalation of 5% of isoflurane followed by cervical dislocation. Tumors were subsequently surgically removed for further histopathological analysis.

The integrin specificity of ^68^Ga-NODAGA-RGD was assessed in supplementary blocking studies using in vivo PET imaging in five xenograft mice.

Finally, an additional comparison of the PET images provided by ^18^F-FDG and ^68^Ga-NODAGA-RGD was obtained in five rats, after brain stereotactic xenografts of the U87-MG cells, as described previously [15]. Briefly, 8-week-old athymic male nude rats (180–220 g), (Hsd: RH-Foxn1rnu; Envigo, Gannat, France), were anesthetized with a mixture of air and 1.5–2% isoflurane and were placed in a Kopf stereotactic frame (900 M Kopf Instruments, Tujunga, CA). After a scalp incision, a burr hole was drilled 0.5 mm anterior and 2.7 mm lateral to bregma and a skull anchor (Patent N° 11 55596) was affixed. Thereafter, approximately 5.10^4^ U87-MG cells, suspended in 5 μL Hank’s Buffered Salt Solution (HBSS, 1×), were injected into the brain parenchyma at 4.4 mm depth. ^18^F-FDG and ^68^Ga-NODAGA-RGD PET were recorded 48-h apart, and 11 to 14 days after xenograft implantation.

### Small animal PET studies

PET recordings were obtained with a camera dedicated to small animal studies (Inveon, Siemens Preclinical Solutions, Knoxville, USA).

Fasting mice were anesthetized with 1.5% isoflurane after which either 6 MBq of ^68^Ga-NODAGA-RGD or 15 MBq of ^18^F-FDG were injected as a bolus via a lateral tail vein cannulation after determination of baseline glycemia by a dedicated system (Optium Xceed, Abbott, France). List-mode acquisitions of 120 min durations were initiated a few seconds prior to tracer injection, and the acquired PET data were subsequently reconstructed in 27 consecutive frames (i.e., 5 frames of 120-s duration followed by 22 frames of 5-min duration) using the ordered-subsets expectation maximization 3D algorithm (OSEM3D, 4 iterations, 16 subsets, zoom 1) together with scatter and attenuation corrections based on transmission source measurement. The final voxel size was 0.8 × 0.8 × 0.9 mm^3^.

Tumor and of various organ activities were determined through SUV mean values within 3D regions of interests (ROI), which were drawn with a dedicated software (Inveon Research Workplace 4.1, Siemens®, Knoxville, USA) on the fusion images encompassing the entire 120-min recording period. Spheroid ROIs were placed inside the liver, brain, and kidney regions with the ROI limits approximating the organ limits as close as possible. Tumor and heart ROIs were obtained with isocontour ROIs and threshold limits of 50 and 80%, respectively, of the maximal voxel value. In this manner, no difference in ROI volumes was documented between ^68^Ga-NODAGA-RGD and ^18^F-FDG.

For the blocking studies performed in five additional xenograft mice, a native PET was recorded with ^68^Ga-NODAGA-RGD (5.9 ± 0.30 MBq) as described above, and this protocol was repeated unchanged 2 days later, except that ^68^Ga-NODAGA-RGD was co-injected with a saturating dose of unlabeled RGD (26 ± 3 mg kg^−1^ of c(RGDyK)). The tracer uptake was calculated through SUVmean values within the aforementioned described ROIs.

The ^68^Ga-NODAGA-RGD and ^18^F-FDG PET study protocols were unchanged for the rat experiments, aside from (i) higher activities were injected (approximately 40 MBq for ^68^Ga-NODAGA-RGD and 74 MBq of ^18^F-FDG), (ii) the PET recording was focused on the head area, and (iii) tumor activities, determined during the 2^nd^ hour following tracer injection, were expressed relative to the activities from the contralateral normal brain (i.e., with the tumor ROI being duplicated and then moved to the contralateral area).

### Gamma counter-based biodistribution study

Biodistribution was additionally analyzed ex vivo in tumor-bearing mice and for both ^68^Ga-NODAGA-RGD and ^18^F-FDG. Seventeen fasting mice were anesthetized with 1.5% isoflurane after which, 6 MBq of ^68^Ga-NODAGA-RGD (*n* = 8) or 15 MBq of ^18^F-FDG (*n* = 9) was injected as a bolus via a lateral tail vein. Animals were sacrificed by cervical dislocation 60 min after injection. Various tissues (tumor, liver, heart, brain, and kidney) were dissected, weighed, and counted in a calibrated gamma counter (Wizard, PerkinElmer, France). The results were expressed as percentages of injected dose and per gram of tissue (% ID/g).

### Tissue sampling, autoradiography, and histology

Immediately after the last PET imaging protocol, the anesthetized animals were sacrificed by cervical dislocation. Tumors were subsequently removed surgically, and tissue samples were frozen in isopentane mixed with dry ice and sectioned in serial 20 and 8 μm sections for autohistoradiography and immunohistochemical staining, respectively. The distribution of ^68^Ga-NODAGA-RGD activity in the tumor sections was recorded with an autohistoradiography system dedicated to the detection of electrons and positrons (μImager™, Biospace, France). For further histopathological examination, adjacent tumor sections were fixed with paraformaldehyde 4%, stained with hematoxylin and eosin or with a specific rabbit polyclonal antibody to determine the expression of human αv integrin (anti-CD51, Cloud-Clone Corporation, PAB282Hu01) and Ki-67 protein (Thermo Fischer SP6 MAS-14520). Prior to incubation with antibodies, the tumor sections were incubated with a citrate buffer (pH 6.0) for 35 min at 97 °C.

### Statistical analyses

All data are expressed as mean ± SEM. Statistical analyses were performed with SPSS Statistics Software v. 20 (IBM, NY, USA). Paired and unpaired comparisons of quantitative variables were performed with Student’s *t* tests and ANOVA tests, respectively. *P* values less than 0.05 were considered as statistically significant.

## Results

### Radiochemistry

An automated ^68^Ga-labeling of NODAGA-RGD resulted in a high labeling efficiency (> 98%) with a specific activity ranging from 20 to 30 MBq/nmol. The overall process, including synthesizer test, cassette test, radiosynthesis, and quality control leading to the production of around 250 MBq of ^68^Ga-NODAGA-RGD, lasted approximately 60 min. Radiochemical purity determined by HPLC was always > 95%.

### PET imaging and biodistribution studies

**Fig. 1:**
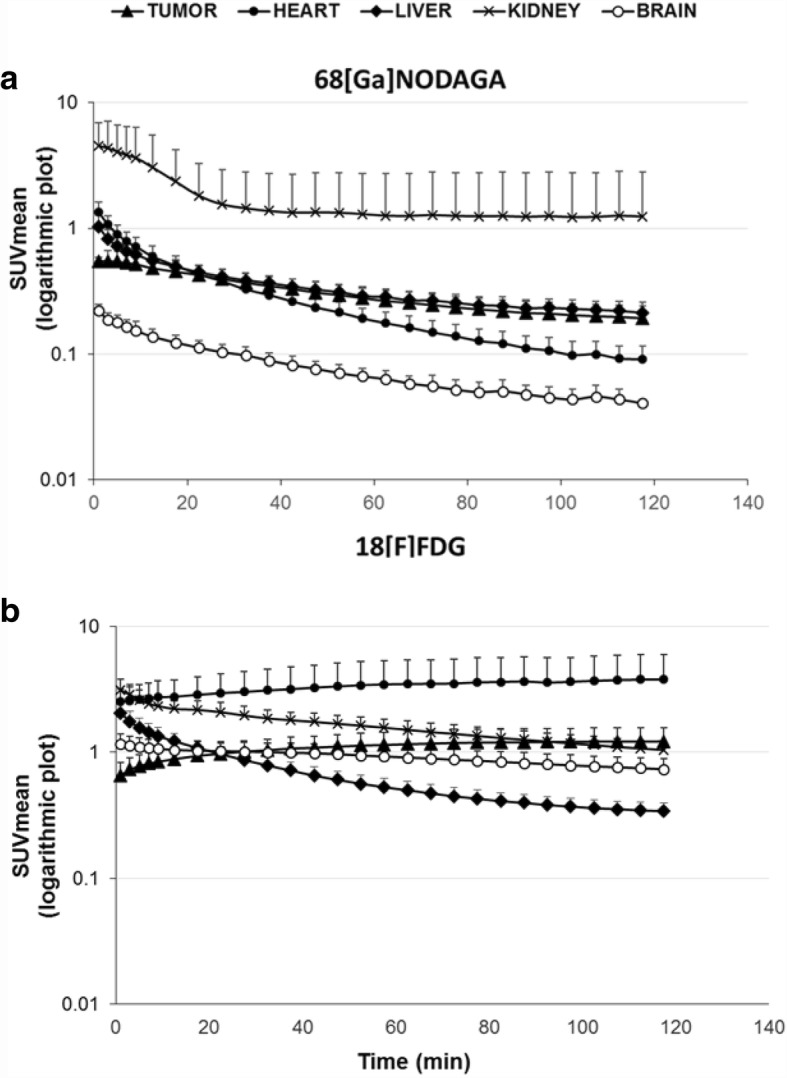
Time-activity-curves derived from the 120-min PET recordings following the intravenous injection of ^68^Ga-NODAGA-RGD (**a**) or ^18^F-FDG (**b**) (*n* = 17 for both tracers) and expressed after logarithmic transformation of the mean ± SEM values for the SUVmean from the kidneys (black crosses), tumor (black triangles), heart (black circles), brain (white circles), and liver (white squares)

The time-activity-curves (TACs), obtained in vivo for the tumors and for different organs after the injection of ^68^Ga-NODAGA-RGD or ^18^F-FDG on whole-body PET images of engrafted mice, are shown in Fig. 1. Both ^68^Ga-NODAGA-RGD and ^18^F-FDG showed a primary renal route of tracer clearance, although this clearance was faster for ^68^Ga-NODAGA-RGD resulting in particularly high activities in the kidneys and bladder. Tumor uptake of ^68^Ga-NODAGA-RGD decreased progressively with time, contrary to that of ^18^F-FDG for which a progressive enhancement was documented throughout the 2 h of analysis.

At 60 min post-injection, the mean tumor activity of ^68^Ga-NODAGA-RGD (SUVmean 0.23 ± 0.04) was one-fifth of that documented for ^18^F-FDG (1.19 ± 0.33; *p* < 0.01). By contrast, the TAC levels of the liver were comparable for the two tracers, and, as expected, heart and brain TAC levels were much higher for ^18^F-FDG (Fig. 1).

**Fig. 2:**
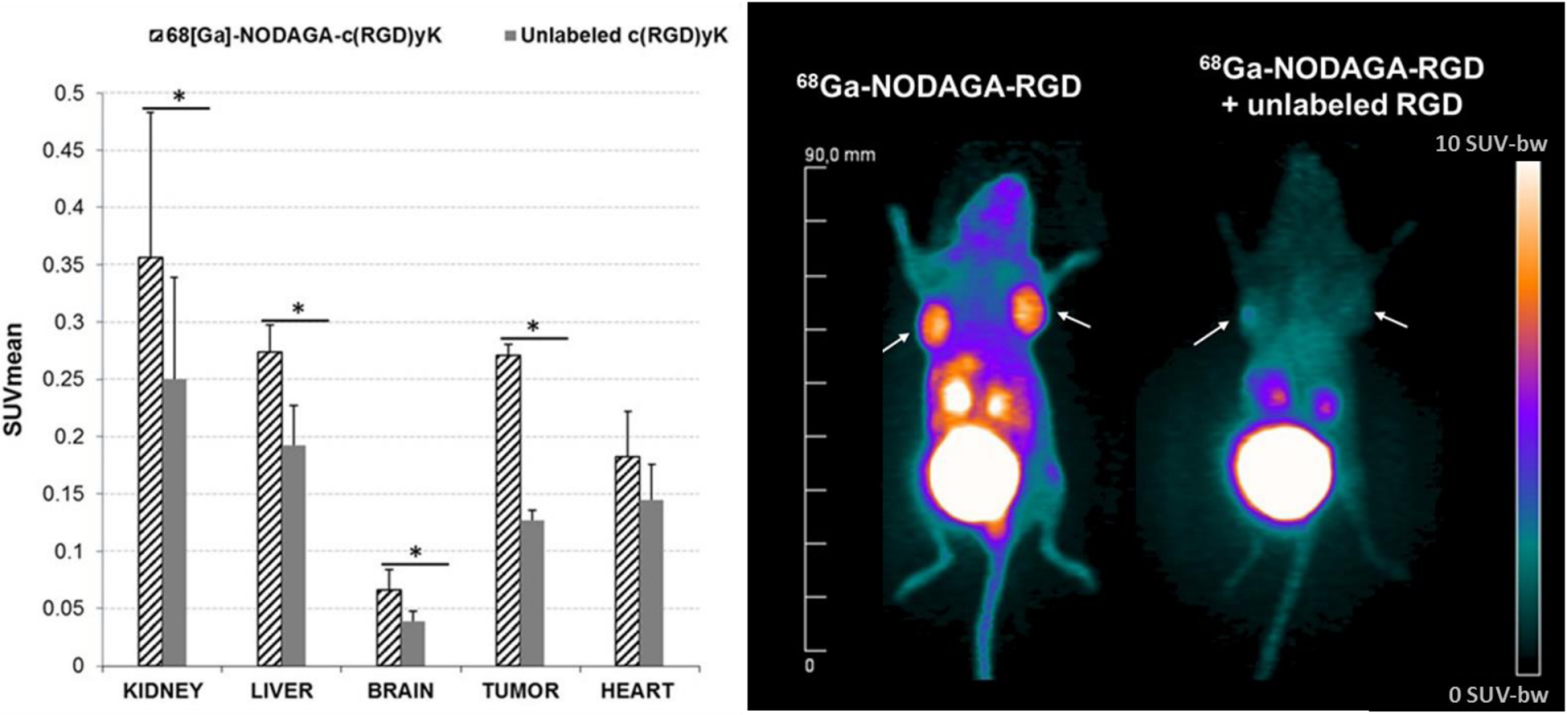
Results of blocking experiments performed in five xenograft mice: **a** with SUVmean values obtained at the 2^nd^ hour of PET recording for tumors and various organs, after the injection of ^68^Ga-NODAGA-RGD (black hatched bar), as compared with the co-injection of ^68^Ga-NODAGA-RGD with excess unlabeled RGD (gray bars) and **b** with examples of superimposed Maximal Intensity Pixel images obtained in the same animal with ^68^Ga-NODAGA-RGD alone (left image of mice) and with ^68^Ga-NODAGA-RGD co-injected with unlabeled RGD (right image of mice). The two tumors are indicated with white arrows and the color scale is expressed in SUV values. *p < 0.05 for paired comparisons

As detailed in Fig. 2, the tumor activity from ^68^Ga-NODAGA-RGD, which likely corresponded to true integrin binding (i.e., suppressed by the co-injection of a saturating excess of unlabeled RGD), was found relatively high, although only at the 2^nd^ hour following injection, corresponding on average to 53% of the total tumor activity. By contrast, this saturation-related decrease was much lower (around 25%, on average), when computed in the 1^st^ hour of PET recording.

As additionally shown in Fig. 2, a significant saturation-related decrease in ^68^Ga-NODAGA-RGD uptake was also observed on most of the other analyzed organs (liver, brain, and kidneys), although the amplitudes of these decreases were much lower than that observed for the tumor.

**Fig. 3:**
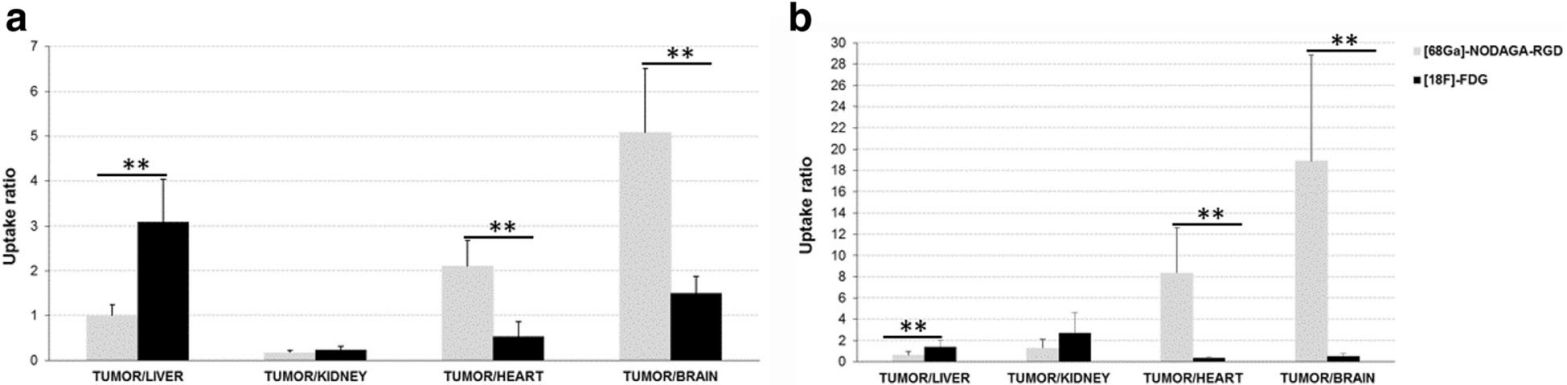
Comparison of tumor-to-organ ratios for activities measured during the 2^nd^ hour with PET recording (**a**) and gamma counter (**b**) between ^68^Ga-NODAGA-RGD and ^18^F-FDG in 17 xenograft mice (**p < 0.001 for ^68^Ga-NODAGA-RGD *vs.* ^18^F-FDG)

As detailed in Fig. 3, activity ratios between the tumor and various organs were computed for the second hour of PET recording and gamma counting, as well as in a comparative manner between ^68^Ga-NODAGA-RGD and ^18^F-FDG. These ratios were found lower with ^68^Ga-NODAGA-RGD than with ^18^F-FDG for tumor-to-liver but higher for tumor-to-heart or tumor-to-brain ratios. On average, with PET imaging, the tumor-to-normal brain ratio was 3.4 times higher for ^68^Ga-NODAGA-RGD (5.1 ± 1.4) than for ^18^F-FDG (1.5 ± 0.37) (Fig. 3a). This ratio was furthermore higher when computed with ex vivo biodistribution data (19.8 ± 10 vs. 0.61 ± 0.2%ID/g) for ^68^Ga-NODAGA-RGD and ^18^F-FDG, respectively (Fig. 3b).

**Fig. 4:**
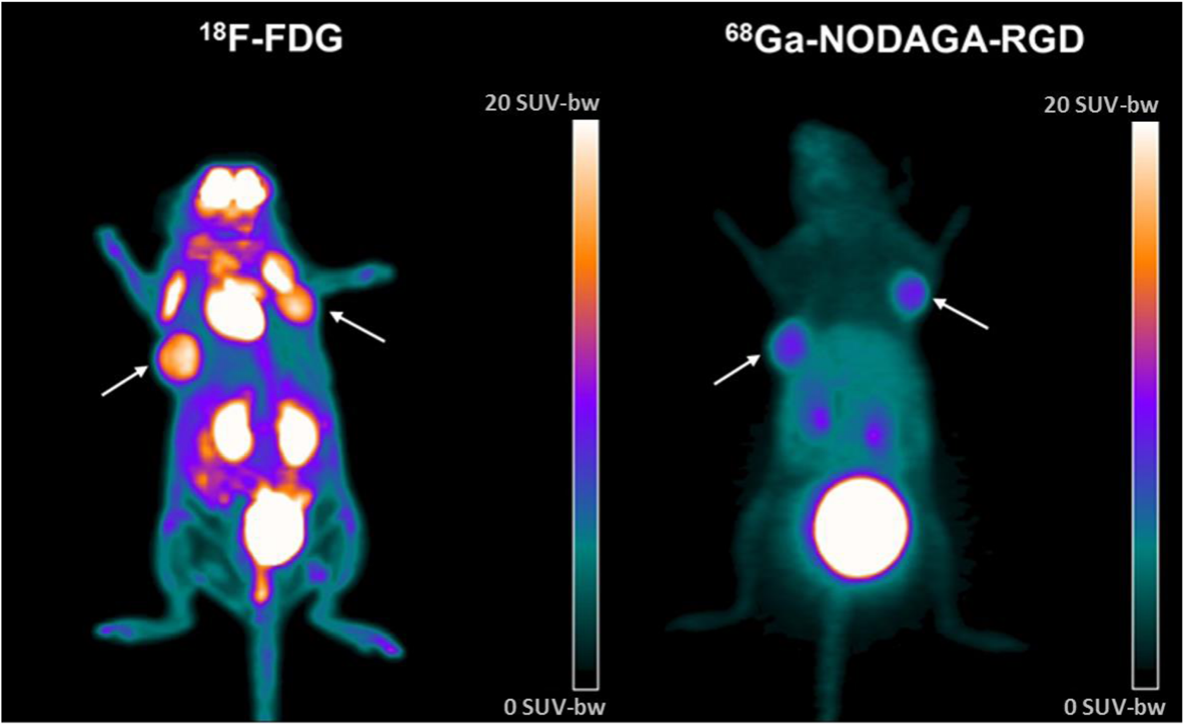
Example of superimposed Maximal Intensity Pixel images corresponding to the 2^nd^ hour of PET recording and obtained in the same xenograft mouse with ^18^F-FDG (left panel) and with ^68^Ga-NODAGA-RGD (right panel). The two tumors are indicated with white arrows, and the color scale is expressed in SUV values

Whole-body images of the 2nd hour of PET recordings are depicted in Fig. 4 for both tracers, providing an illustration of the potential of ^68^Ga-NODAGA-RGD for tumor imaging of thoracic and head areas, with much lower normal uptakes observed for ^68^Ga-NODAGA-RGD than for ^18^F-FDG in these areas.

**Fig. 5:**
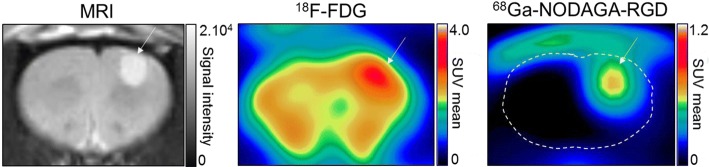
Example of magnetic resonance images and PET images obtained with ^18^F-FDG and with ^68^Ga-NODAGA-RGD in a rat brain with an engrafted glioblastoma tumor (white arrows)

A further illustration of the higher potential of ^68^Ga-NODAGA-RGD for the imaging of brain glioblastomas is provided in Fig. 5, from PET images recorded during the 2nd hour in a rat with an engrafted brain tumor. On average, in the five nude rats with engrafted glioblastomas, tumor activity expressed relative to that of the contralateral normal brain was found to be 3.73-fold higher for ^68^Ga-NODAGA-RGD (6.05 ± 0.67) than for ^18^F-FDG (1.62 ± 0.15) (see individual data in the Additional file 1: Table S1).

### Ex vivo analyses

**Fig. 6:**
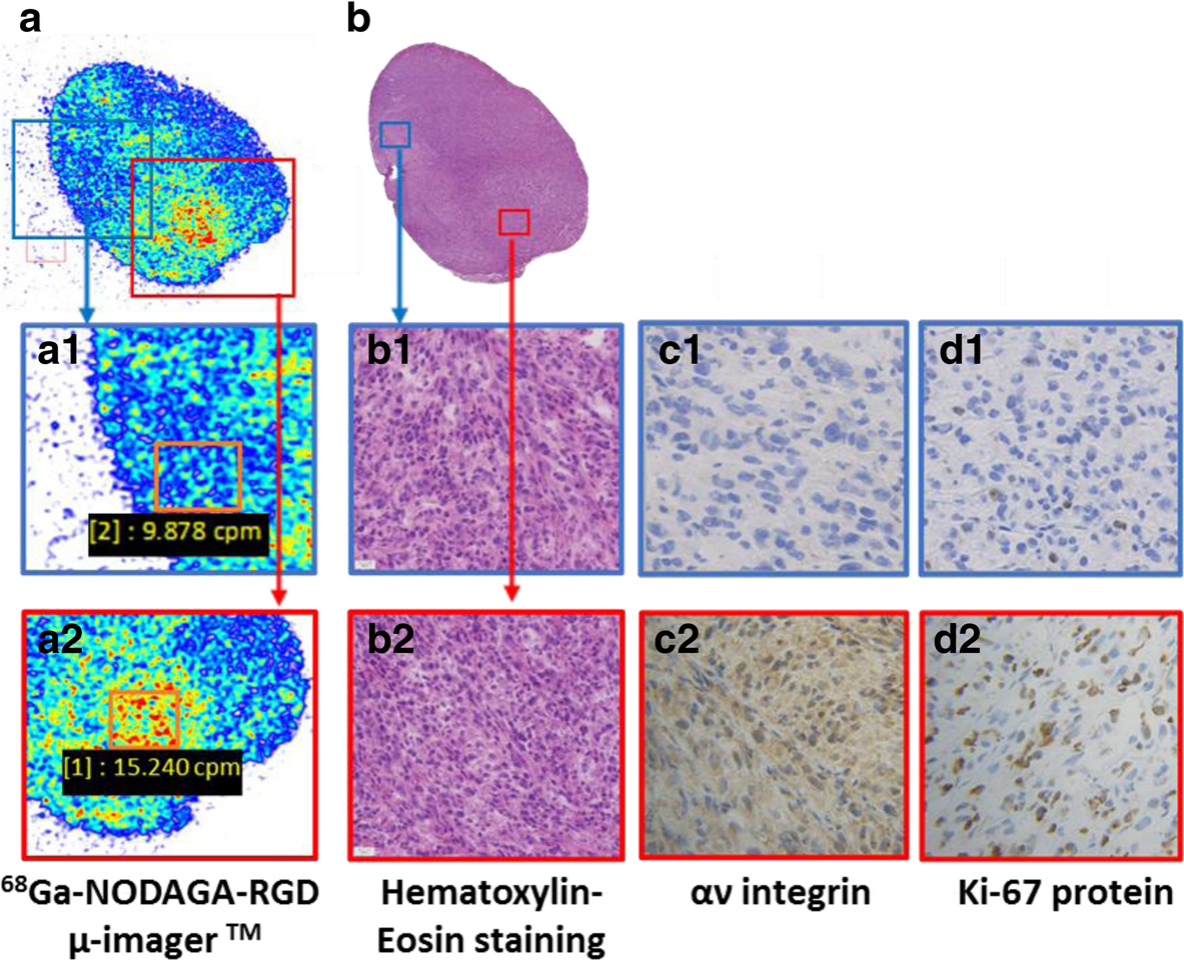
Representative examples of ex vivo images obtained from the same glioblastoma tumor extracted from a nude mouse after sacrifice showing (1) the distribution of ^68^Ga-NODAGA-RGD recorded by the μImager™ and (2) with several immunohistochemical staining applied on contiguous slices for a morphological analysis (hematoxylin-eosin) and for analyzing the expressions of the αv subunit and the Ki-67 protein (an indicator of cell proliferation). Note that the expressions of the αv subunit (third panel c2 bottom) and of the Ki-67 (last panel bottom d2 right) protein appear much higher in a selected area showing a high level of recorded ^68^Ga-NODAGA-RGD counts on the μImager™ (15.240 counts-per-minute, images from the lower third of the figure; first panel bottom left), when compared with another area showing a low level of recorded counts (9.878 counts-per-minute, images from the middle third of the figure; first panel middle). By contrast, cell density appears equivalent in these two areas (second panel b1 and b2 middle and bottom) on the hematoxylin-eosin stained images

A representative example of the ex vivo analyses obtained after the injection of ^68^Ga-NODAGA-RGD is given in Fig. 6. Autohistoradiographic analyses with the μImager™ revealed a heterogeneous intratumoral distribution of ^68^Ga-NODAGA-RGD; of note, areas exhibiting a higher ^68^Ga-NODAGA-RGD activity were also those showing higher expressions of the integrin αv subunit and of the protein Ki-67, an indicator of cell proliferation. By contrast, in hematoxylin-eosin stained tissue slices, cells and nuclear densities were comparable between tumor areas with and without high ^68^Ga-NODAGA-RGD uptake (Fig. 6).

## Discussion

In this experimental study, the kinetics and biodistribution of ^68^Ga-NODAGA-RGD, a monomeric RGD-containing tracer, were found to be more favorable for the PET imaging of glioblastoma than those of ^18^F-FDG, a reference tracer in oncology. Much higher tumor-to-normal brain ratios were indeed observed in two distinct rodent models of engrafted human U87-MG cells, one with tumors engrafted in mouse flanks and the other with direct engraftment in the rat brains.

The rationale behind these observations is that αvβ3 integrins, which are targeted by RGD-containing tracers, are highly expressed in high-grade gliomas in which the magnitude of this expression has been associated with the potential for tumor invasion, angiogenesis, and metastasis [6, 7, 16–19]. Furthermore, these integrins are known to be poorly expressed within the different tissue components of normal brain and, in addition, the RGD-containing tracers may pass through the blood brain barrier when disrupted by a pathological process [8, 18–22]. These considerations likely explain the high tumor-to-normal brain ratios documented herein for ^68^Ga-NODAGA-RGD, i.e., approximately five for the mouse model and six for the rat model, on average. This ratio was conversely much lower for ^18^F-FDG, i.e., approximately 1.5 for the mouse model and 1.6 for the rat model on average, owing to the variable glucose metabolism of high-grade gliomas [23] in addition to the high residual glucose metabolism of normal brain cells.

In further in vivo PET experiments, a seemingly high level of integrin binding of ^68^Ga-NODAGA-RGD within the tumors was documented by blocking experiments (i.e., with inhibition of integrin binding in the presence of saturating doses of unlabeled RGD). It should be pointed out, however, that this proportion of integrin binding was sufficiently high, i.e., corresponding to more than 50% of tumor activity measured on PET images, only during the second hour of the PET recording. This observation is in accordance with previous literature on RGD-containing tracers, leading to the current recommendation of delaying PET recording for 30 to 60 min after tracer injection [3, 10, 11]. This allows avoiding an early high level of nonspecific activity, possibly due to the circulating blood tracers, and leading to overestimate integrin density.

A significant saturation-related decrease in ^68^Ga-NODAGA-RGD uptake was also observed on most of the other analyzed organs, including the brain, although the amplitude of this decrease was much lower than for the tumor. Similar observations have also been reported for other RGD-containing radiotracers in both humans and animals [24–28], owing to the fact that integrin αvβ3 is also expressed in various normal cells, including vascular smooth muscle cells, macrophages, and osteoclasts.

Further immunohistological analysis indicated a rather good correspondence between tumor sites, showing the highest level of ^68^Ga-NODAGA-RGD activity as well as high levels of αv integrin expression (Fig. 6), confirming the integrin specificity of this tracer. In these ex-vivo analyses, the heterogeneous tumor level of ^68^Ga-NODAGA-RGD activity was found to have a greater correspondence with the local distribution of cell proliferation (Ki-67 protein expression) rather than that of cell density. These observations further strengthen the general consideration that RGD-based radiotracers would be particularly indicated for characterizing high-grade gliomas showing high potential for tumor invasion, angiogenesis, and metastasis, as stated above.

On the other hand, these characteristics are likely to differ for ^18^F-FDG which rather reflects the distribution of glucose uptake and subsequent phosphorylation by the tumor cells. This may explain the lack of correlation noted herein between tumor uptake of ^18^F-FDG and that of ^68^Ga-NODAGA-RGD (results not shown), in accordance with a previous comparison performed with ^18^F-galacto-RGD in men with various cancer types [9, 29, 30].

^18^F-galacto-RGD is another monomeric RGD tracer, for which tumor uptake has been shown to be closely related to αvβ3 integrin expression, and was the first reported RGD-based tracer in human subjects [9, 31]. Unfortunately, the ^18^F-labeling of this tracer and of all RGD-containing peptides appears to be too complex for routine application [12]. RGD-containing peptides, labeled with ^68^Ga by a current metal complexation process, are conversely much easier to, synthesize.

This is particularly the case for our automated ^68^Ga-labeling process of NODAGA-RGD, resulting in a > 98% labeling efficiency and > 95% radiochemical purity. In a more general manner, the generator-produced ^68^Ga represents an attractive alternative to cyclotron-produced radionuclides in spite of a slightly lower image quality than that provided by ^18^F (owing to a higher energy of the emitted positron).

Our small monomeric RGD-containing tracer exhibited relatively rapid kinetics with a primary renal route of tracer clearance, which was faster than that of ^18^F-FDG, resulting in higher activities in the kidneys and bladder. It is likely that these somewhat high activities documented in the kidneys, bladder, and to a lesser extent in the liver may hamper imaging of abdominal tumors, as previously observed for other RGD-containing radiotracers [3, 10], although would likely not constitute a significant limitation for brain tumor imaging.

The fast-renal clearance of ^68^Ga-NODAGA-RGD was associated however, with a progressive decline in tumor activity with time, contrary to the progressive increase documented for ^18^F-FDG, which may constitute a limitation for brain imaging of high-grade gliomas. Taken together, as a result of this rather fast tumor clearance and the constraint of delaying PET recording to the 2nd hour following injection, dedicated clinical studies are thus mandatory to accurately ascertain the required level of activity to be injected for the late imaging of a sufficiently high tumor activity.

Additional comparisons are also required with amino acid PET tracers, such as ^11^C-methionin, ^18^F-FDOPA, and ^18^F-FET, as well as with dimeric RGD-containing tracers, which could provide an enhanced affinity for αvβ3 integrins though a slower diffusion rate when compared with monomeric forms [5].

## Conclusions

This study confirms that ^68^Ga-NODAGA-RGD is a RGD compound with a high ease of synthesis and relatively rapid kinetics. Furthermore, experiments performed in two different rodent models of engrafted glioblastoma give evidence of a high potential for PET imaging, with very high tumor-to-normal brain activity ratios and a triggering of tumor areas with high integrin expression and high cell proliferation rates. However, it is likely that PET recording needs to be delayed until the 2nd hour following injection in order to yield sufficiently specific information on integrin density.

## Additional file

Additional file 1: **Table S1.** Individual results obtained with ^68^Ga-NODAGA-RGD or ^18^F-FDG for the SUVmean values from tumors and contralateral normal brain, as well as for tumor-to-contralateral normal SUVmean ratios. These data were obtained during the 2^nd^ hour of PET recording in five rats with engrafted brain tumors. (DOCX 19 kb)
