# Supplementary material for: PET imaging of 68Ga-NODAGA-RGD, as compared with 18F-fluorodeoxyglucose, in experimental rodent models of engrafted glioblastoma
Source: EJNMMI Res. 2018 Jun 15;8:51. doi: 10.1186/s13550-018-0405-5 (PMC6003898; doi:10.1186/s13550-018-0405-5)
Supplement: Supplementary file 1 — Table S1. Individual results obtained with 68Ga-NODAGA-RGD or 18F-FDG for the SUVmean values from tumors and contralateral normal brain, as well as for tumor-to-contralateral normal SUVmean ratios. These data were obtained during the 2nd hour of PET recording in five rats with engrafted brain tumors. (DOCX 19 kb) [file 13550_2018_405_MOESM1_ESM.docx]

Additional file 1

|  | ^68^[Ga]-NODAGA-RGD | | | | | | | |  | ^18^[F]-FDG | | | | | | | |
| --- | --- | --- | --- | --- | --- | --- | --- | --- | --- | --- | --- | --- | --- | --- | --- | --- | --- |
|  | SUVmean_Tumor | | |  | SUVmean_ contralateral normal brain | | | Tumor-to-contralateral normal brain ratio |  | SUVmean_Tumor | | |  | SUVmean_ Contralateral normal brain | | | Tumor-to-Contralateral normal brain ratio |
| Rat 1 | 0.5 | ± | 0.01 |  | 0.1 | ± | 0.01 | 5.47 |  | 2.9 | ± | 0.06 |  | 1.7 | ± | 0.2 | 1.36 |
| Rat 2 | 0.7 | ± | 0.03 |  | 0.1 | ± | 0.02 | 5.65 |  | 3 | ± | 0.08 |  | 2 | ± | 0.21 | 1.60 |
| Rat 3 | 0.5 | ± | 0.01 |  | 0.1 | ± | 0.01 | 5.36 |  | 4.7 | ± | 0.14 |  | 3.1 | ± | 0.27 | 0.63 |
| Rat 4 | 0.6 | ± | 0.02 |  | 0.1 | ± | 0.01 | 6.38 |  | 5.2 | ± | 0.08 |  | 2.7 | ± | 0.27 | 1.31 |
| Rat 5 | 0.6 | ± | 0.01 |  | 0.1 | ± | 0.01 | 7.40 |  | 4.2 | ± | 0.07 |  | 2.9 | ± | 0.35 | 1.24 |

Table S1. Individual results obtained with ^68^Ga-NODAGA-RGD or ^18^F-FDG for the SUVmean values from tumors and contralateral normal brain, as well as for tumor-to-contralateral normal SUVmean ratios. These data were obtained during the 2^nd^ hour of PET recording in 5 rats with engrafted brain tumors.
